# Effect of Powder/Water Ratio Variation on Viscosity, Tear Strength and Detail Reproduction of Dental Alginate Impression Material (*In Vitro* and Clinical Study)

**DOI:** 10.3390/polym13172923

**Published:** 2021-08-30

**Authors:** Rasha M. Abdelraouf, Rania E. Bayoumi, Tamer M. Hamdy

**Affiliations:** 1 Biomaterials Department, Faculty of Dentistry, Cairo University, Cairo 11553, Egypt; 2 Biomaterials Department, Faculty of Dentistry (Girls), Azhar University, Cairo 11754, Egypt; raniaezzat.26@azhar.edu.eg; 3 Restorative and Dental Materials Department, National Research Centre (NRC), Giza 1226, Egypt; dr_tamer_hamdy@yahoo.com

**Keywords:** polymeric alginate impressions, powder/water ratio, viscosity, tear strength, detail reproduction

## Abstract

Background: Alginate impression is a common dental polymeric material, presented as powder to be mixed with water. Aim: 1. To analyze the effect of alginate powder/water ratio variation on viscosity, tear strength and detail reproduction by *in vitro* tests, and 2. To evaluate this variation’s effect on patients’ impressions. Materials and methods: Two commercial alginate products were mixed in different viscosities. Viscosity was measured by a viscometer. For the tear strength test, V-shaped specimens were used. For detail reproduction, a die with three scribed lines was used. Clinical dental impressions were examined by stereomicroscope. Results: The alginate specimens mixed with a higher powder/water ratio showed a higher viscosity and tear strength compared to those with a lower powder/water ratio. Both alginate mixtures reproduced two scribed lines in a detail reproduction test. On the other hand, no clear clinical difference was detected when examining dental impressions mixed with a different powder/water ratio. Conclusion: Although increasing the powder/water ratio of mixed alginate raised the resultant viscosity and tear strength by an *in vitro* test, clinically, no clear difference in tearing was detected. Detail reproduction was minimally affected by the variation in powder/water ratio.

## 1. Introduction

Alginate impression materials are one of the most widely used materials in dental clinics [1]. Alginates are the salts of alginic acid, which is a polysaccharide derived from marine algae [1]. Potassium or sodium alginates are used in the dental field as they are water soluble and react with calcium ions, forming an insoluble calcium alginate gel [1]. Fillers such as diatomaceous earth are added to the dental alginate to strengthen the gel.

Once the dental alginate powder is mixed with water, the alginate is changed into a soft paste (sol) that is converted to a gel by two chemical reactions: the first is a retardation reaction, giving time for the alginate to be manipulated and inserted into the patient’s mouth, and the second is a gelation reaction [1]. In the retardation reaction, a retarder (sodium phosphate) reacts preferentially with a reactor (calcium sulfate) to provide working time before gelation [1]. The second reaction is gelation, where the soluble salts of alginic acid (potassium or sodium alginate) react with the calcium ions released from the reactor, forming an insoluble calcium alginate gel [1].

Dental alginate is presented as a powder to be mixed with water in ratios that have been proportioned by the manufacturers [2]. Certain measures, such as a scoop for the powder and a graduated beaker for the water, are provided with each commercial product for easier manipulation [2]. It was noticed that the powder/water ratio varies among the different companies, resulting in obvious variation in the viscosity of the produced mix, being thick and with high viscosity in some products, while others have a lower viscosity [3]. This may affect the properties of the set alginate [3]. The tear strength is very critical in this hydrocolloid material as it suffers from low tear strength [4]. In addition, detail reproduction is a crucial requirement for any dental impression material [5].

Although several research reports compare the tear strength and detail reproduction of multiple commercial alginate products [4,5,6,7], there is not enough evidence about the effect of manipulative variables on the alginate’s properties [8,9]. The simplicity of hand mixing the alginate may be a double-edged weapon [9]. This facilitates its manipulation with minimal equipment; thus, hand mixing is very common compared to mechanical mixing [9]. In contrast, hand mixing may introduce several manipulative variables, for example, the operator may change the powder/water ratio from that recommended by the manufacturer [8].

Thus, there are two main issues which may concern dentists when mixing alginate powder with water. The first issue is the great variation in the alginate’s consistency and powder/water ratio among the different commercial products. Some products produce a flowy thin mix after mixing, while others give a thick viscous mix. Some dentists think that a thick viscous mix increases the tear strength, so they prefer products which produce a thick viscous consistency, while others prefer a thin flowy mix, aiming for better detail reproduction. The first research question is: does the alginate viscosity actually affect tear strength and detail reproduction? The second issue is that some dentists do not follow manufactures’ instructions in mixing. They rather prefer to perform the mix using their own preference and experience. They deviate from the recommended ratio by increasing or decreasing the powder/water ratio. So, the second part of the research studies the effect of powder/water variation by 25% (which still gives a workable mix) on tear strength and detail reproduction.

There was a deficiency in the literature about the effect of powder/water ratio variation and its resultant viscosity upon tear strength and detail reproduction of dental alginate impression materials. In addition, no previous studies clinically examined the impressions taken from patients’ mouths and correlated these results with those obtained by an *in vitro* laboratory test.

Therefore, this research assesses the effect of powder/water ratio variation on viscosity, tear strength and detail reproduction of dental alginate impression material by *in vitro* tests. In addition, dental impressions with different viscosities are taken clinically from volunteer dental patients and the resultant impressions are examined with a stereomicroscope.

## 2. Materials and Methods

Two commercial dental alginate impression materials were used in this study as listed in Table 1: "Algistar" (Müller-Omicron GmbH & Co. KG, Lindlar, Germany) and "Tropicalgin" (Zhermack Spa, Italy).

### 2.1. Specimen Grouping

The specimens were divided into two main groups according to the type of alginate impression used: (1) Algistar; (2) Tropicalgin. Each group was further sub-divided into two sub-groups according to powder/water ratio: (I) According to manufacturer’s instructions; (II) Variation in powder weight (wt.) by 25%.

Thus, the four groups were as follows (Table 2):

Group 1: Algistar mixed according to the manufacturer’s instructions (19 g powder/40 mL water). It was observed that the resultant mix had a flowy consistency.

Group 2: Algistar mixed with a higher powder/water ratio than that recommended by the manufacturer (23.75 g powder/40 mL water), i.e., powder wt. increased by 25%. This resulted in a thicker mix consistency than the previous group.

Group 3: Tropicalgin mixed with a lower powder/water ratio than that recommended by the manufacturer (13.5 g powder/36 mL water). It was observed that mixing Tropicalgin alginate following the manufacturer’s instructions resulted in a thick consistency. In this group the powder wt. was reduced by 25%, thus the resultant mix had a more flowy consistency than the one mixed according to the manufacturer’s instructions in the next group.

Group 4: Tropicalgin mixed according to the manufacturer’s instructions (18 g powder/36 mL water).

### 2.2. Specimen Preparation

To prepare the alginate specimens, the powder of each material was weighted using a digital balance (SBA 31, Scaltec, Germany) and then added to the water, which had been measured in milliliters using a graduated test tube and placed in a rubber bowl. Hand mixing was performed using a plastic spatula until a homogenous mix was obtained according to the time recommended by the manufacturer.

### 2.3. Viscosity Measurement (In Vitro)

**Figure 1:**
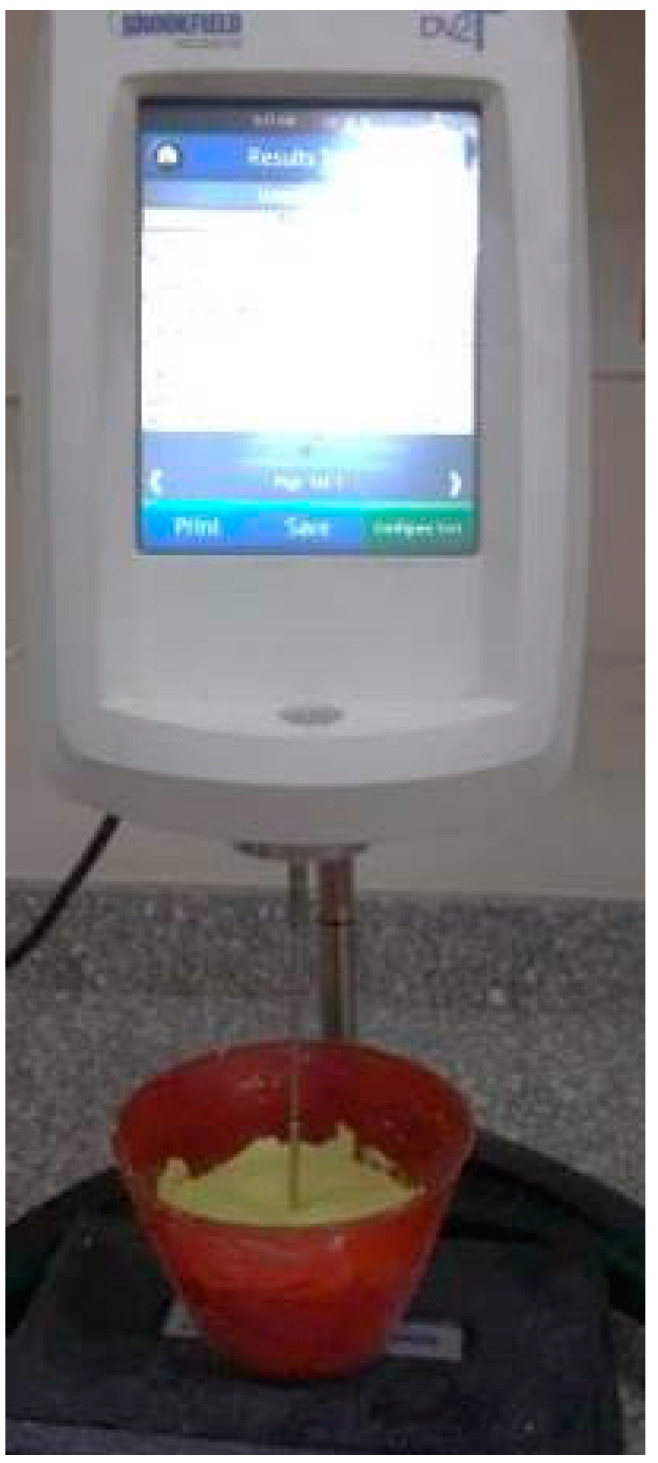
Spindle viscometer.

The viscosity of the mixed alginate was measured by Spindle Viscometer (Ametek Brookfield, Middleboro, MA, USA) as shown in Figure 1, using spindle 64, speed 0.1 rpm at a temperature of 22 °C. The measurement was performed 2 min after the start of mixing in the rubber bowl (n = 6/group). The viscosity was recorded in centipoise (cp).

Statistical analysis of the data was conducted by using the One-Way Analysis of Variance (ANOVA) and Tukey HSD test. The significance level was set to 5% (*p* < 0.05). The data obtained were analysed using IBM® SPSS® Statistics Version 20 for Windows (SPSS Inc., IBM Corporation, Armonk, NY, USA).

### 2.4. Tear Strength Test (In Vitro)

**Figure 2:**
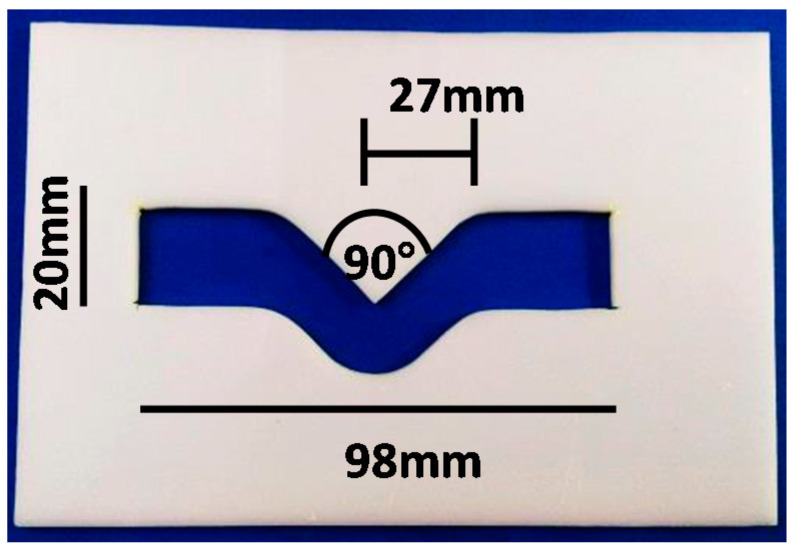
Mold used for tear strength test.

The mixed alginate was poured into a V-shaped Teflon mold specially prepared for tear strength measurement according to the American Society for Testing and Materials, Designation: D 1004-94a. The dimensions of the mold are shown in Figure 2, with a thickness of 4 mm.

**Figure 3:**
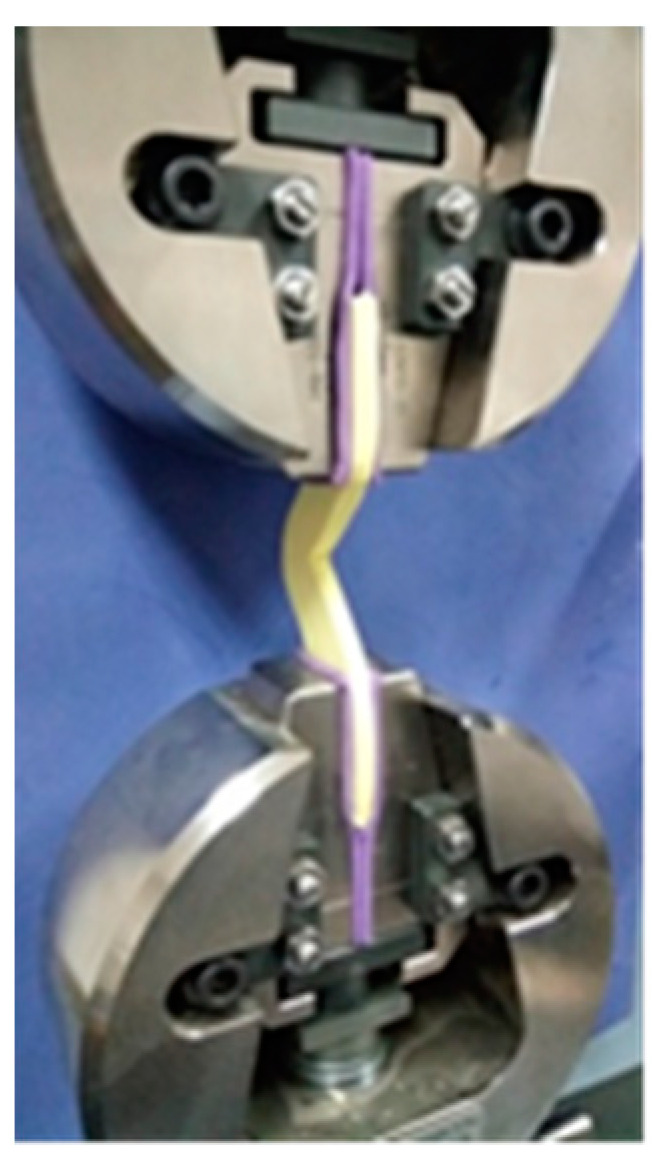
Alginate specimen in Universal Testing Machine.

Two glass slabs were used; one was placed on the bottom of the mold, while the other was positioned upon it. Once the mixed alginate was inserted into the mold, pressure was applied manually until the two glass slabs were in contact with the mold, to ensure even alginate thickness during gelation. After setting was complete, the V-shaped alginate specimens were retrieved out of the mold and immediately subjected to the tear strength test using a universal testing machine (Shimadzu Autograph AG-X plus 5 kN, Kyoto, Japan) as presented in Figure 3, with a crosshead speed of 500 mm/min. The specimens’ tear strength was calculated according to the following equation: Tear Strength = Load required for tearing/Thickness of sample. Statistical analysis was performed as previously described.

### 2.5. Detail Reproduction Assessment (In Vitro)

**Figure 4:**
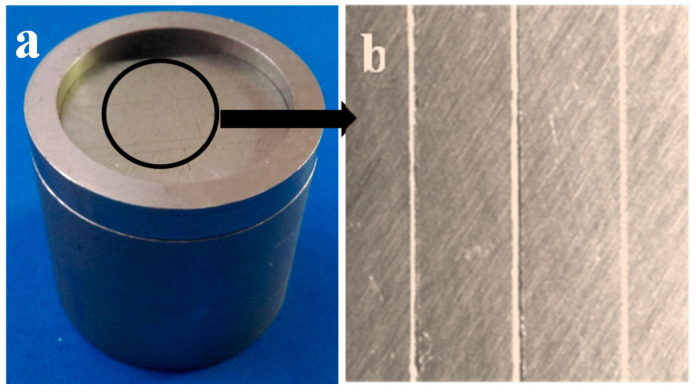
(**a**) Stainless steel die used for detail reproduction assessment, (**b**) Stereomicroscopic images for the three scribed parallel lines.

A stainless-steel die with three scribed parallel lines was used for surface detail reproduction according to the ISO 1563 standard, as shown in Figure 4. The widths of these lines were 20, 50 and 75 μm. A ring was placed on top of the die, and the mixed alginate was loaded inside the ring over the die with the three lines. A glass slab was placed on top of the ring and a one-kilogram weight was positioned upon the slab until setting was complete. Then, the specimens were carefully removed from the mold and immediately examined with a stereomicroscope (Leica, Allendale, NJ, USA) at 10x optical magnification. Specimens were reported to either pass or fail the test based on their ability to capture the entire length of the 50 μm line.

### 2.6. Clinical Dental Impressions

Clinical dental impressions were taken from 18 volunteer dental patients after receiving their informed consents. The inclusion criteria were medically free participants with good oral hygiene. Those with gingivitis or periodontitis were excluded. For each volunteer, four impressions were taken according to the four groups in this study by the same dentist (fifteen years’ clinical experience). All the impressions were removed from the patients’ mouths by sharp snap removal. Then, the impressions were directly examined with a stereomicroscope (Leica, Allendale, NJ, USA) at 10× optical magnification at interproximal contact areas. The main interest was tearing at these areas when the different alginate consistencies were used. In addition, the thickness of the alginate at interproximal contact areas was also measured. Voids in impressions were inspected.

## 3. Results

### 3.1. Viscosity

The mean viscosity values and their standard deviations (SD) are shown in Table 3. There was a significant difference between the viscosities recommended by the manufacturers of the two alginate products (*p* < 0.05). The alginate specimens mixed with a higher powder/water ratio showed a significantly higher viscosity compared to those with a lower powder/water ratio (*p* = 0.0001 *).

### 3.2. Tear Strength

The mean tear strength values and their standard deviations (SD) are shown in Table 4. The alginate specimens mixed with a higher powder/water ratio showed higher tear strength compared to those with a lower powder/water ratio (*p* = 0.0001 *).

### 3.3. Detail Reproduction

**Figure 5:**
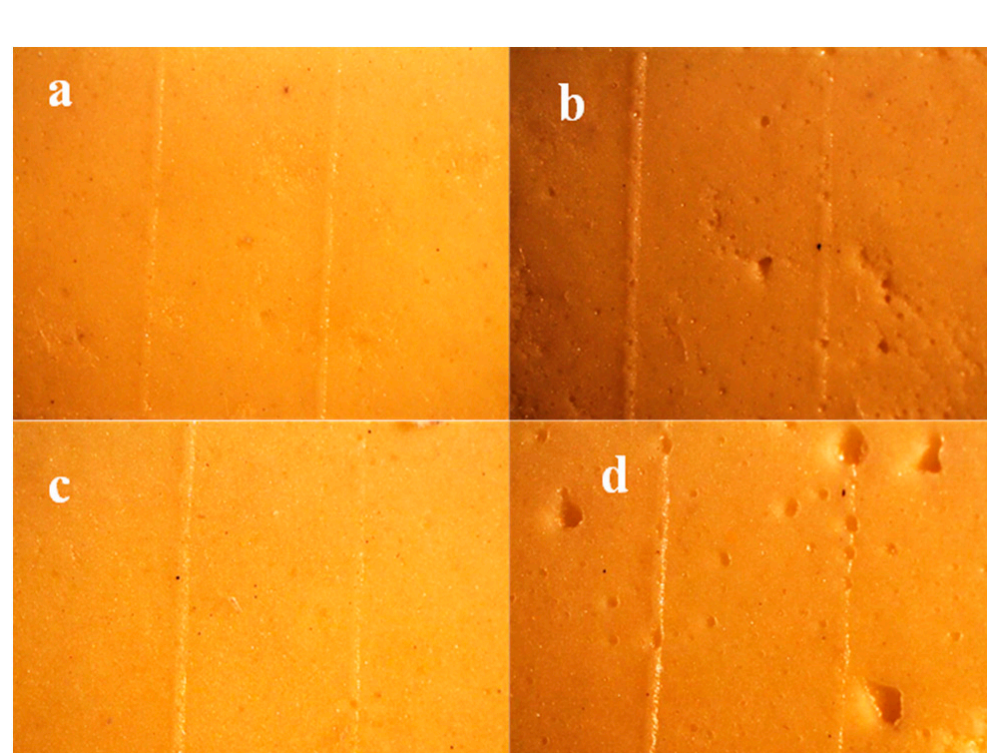
Stereomicroscopic images of specimens assessed for detail reproduction, magnification 10×: (**a**) Group 1 (Algistar mixed as Manufacturer), (**b**) Group 2 (Algistar with ↑Powder/water ratio, (**c**) Group 3 (Tropicalgin with ↓Powder/water ratio), (**d**) Group 4 (Tropicalgin mixed as Manufacturer).

The representative stereomicroscopic images of specimens assessed for detail reproduction are shown in Figure 5a–d.

In all groups, only two lines were reproduced out of the three scribed lines. Those with 50 and 75 μm widths were reproduced while the 20 μm line did not appear. Since detail reproduction was assessed by the alginate impression’s ability to reproduce the entire length of the 50 μm line (ISO 1563 standard), all the specimens passed the test.

It was noticed that there were various porosities and air bubbles in the mixtures of both types with a high powder/water ratio (Figure 5b,d).

### 3.4. Clinical Dental Impression

**Figure 6:**
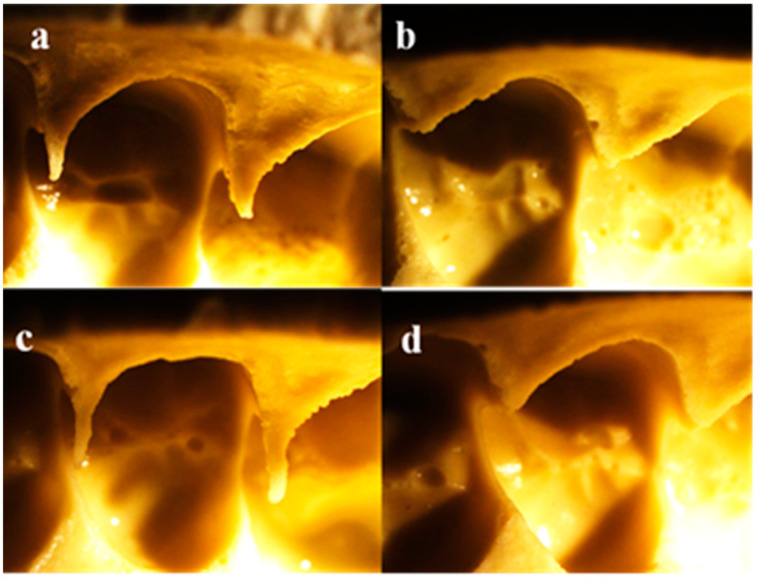
Stereomicroscopic images of clinical impression for patient with excessive proximal contact, magnification 10×: (**a**) Group 1 (Algistar mixed as Manufacturer), (**b**) Group 2 (Algistar with ↑Powder/water ratio, (**c**) Group 3 (Tropicalgin with ↓Powder/water ratio), (**d**) Group 4 (Tropicalgin mixed as Manufacturer).

**Figure 7:**
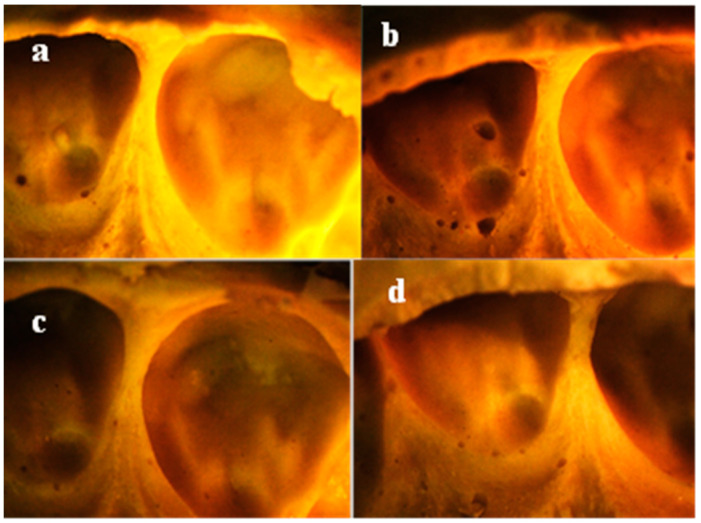
Stereomicroscopic images of clinical impression for patient with open contact, magnification 10×: (**a**) Group 1 (Algistar mixed as Manufacturer), (**b**) Group 2 (Algistar with ↑Powder/water ratio, (**c**) Group 3 (Tropicalgin with ↓Powder/water ratio), (**d**) Group 4 (Tropicalgin mixed as Manufacturer).

**Figure 8:**
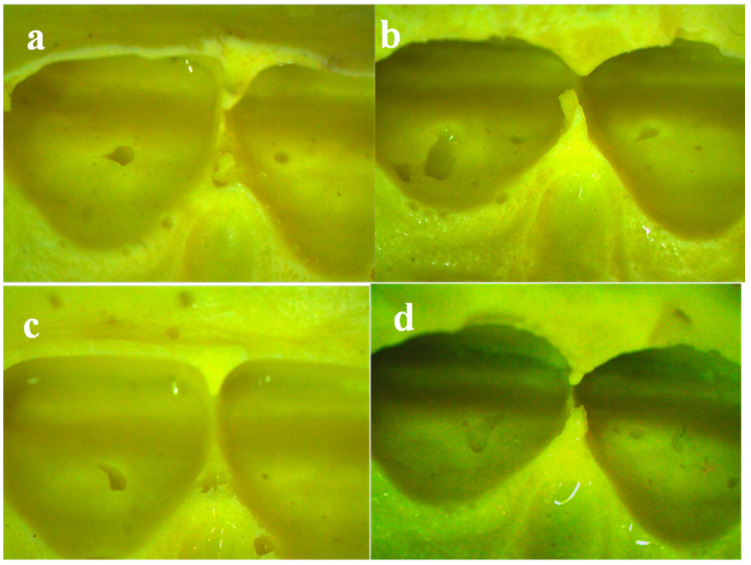
Stereomicroscopic images of clinical impression with proper interproximal contact, magnification 10×: (**a**) Group 1 (Algistar mixed as Manufacturer), (**b**) Group 2 (Algistar with ↑Powder/water ratio, (**c**) Group 3 (Tropicalgin with ↓Powder/water ratio), (**d**) Group 4 (Tropicalgin mixed as Manufacturer).

Representative stereomicroscopic images of clinical impressions from patients are shown in Figure 6, Figure 7 and Figure 8. No clear difference in tearing was clinically detected when examining dental impressions mixed with a different powder/water ratio. It was observed that the tearing of the alginate was greatly affected by the thickness of the impression material at the interproximal area.

In excessive interproximal dental contact areas, the impression materials did not enter between the teeth in all groups tested (Figure 6). Yet, it was noticed that in the area of buccal embrasures, the alginate impressions mixed with a lower powder/water ratio (Figure 6a,c) showed more entrance into the embrasure area compared to those with a higher powder/water ratio (Figure 6b,d).

Meanwhile, in spaced teeth, the impression materials from the different groups entered the open contact areas and were removed from the patients’ mouths without tearing (Figure 7) (mean alginate thickness 1.3 mm ± 0.3).

On the other hand, in proper contact areas, all the impressions were subjected to tearing (Figure 8) (mean alginate thickness 0.5 mm ± 0.2).

Voids were displayed in all patients’ impressions mixed with different powder/water ratios (Figure 6, Figure 7 and Figure 8), yet those mixed with higher powder/water ratios (Figure 6, Figure 7 and Figure 8b,d) showed more voids than those mixed with lower powder/water ratios (Figure 6, Figure 7 and Figure 8a,c).

## 4. Discussion

Making an alginate impression is a common dental procedure [10]. It is presented as a powder to be mixed with water in specific ratios recommended by the manufacturer [2]. It was reported that the resultant mix varied in consistency among the different commercial products [3]. Furthermore, the powder/water ratio may be altered by the operator in the dental clinic according to his clinical preference [11]. It was reported that the prosthodontics judged the preferred alginate viscosity based on their own clinical experience, deviating from the manufacturer’s instructions. As a result, different viscosities were produced, which may affect properties [3] such as tear strength and detail reproduction.

Two commercial alginate products were selected from the dental market. One created a flowy thin mix after mixing (Algistar), while the other produced a thick viscous mix (Tropicalgin). To confirm this observed consistency difference, their viscosities were measured by a viscometer. Comparing their viscosities when mixed as recommended by the manufacturer, there was a significant difference between them. This may be attributed to the difference in the powder/water ratio [11], the molecular weight of the alginate molecule [12,13], the amount of fillers [13] or their size and morphology [14,15].

Being hydrocolloid, the alginate specimens mixed with a higher powder/water ratio were more viscous compared with those with a lower powder/water ratio. This may be due to the reduction of the water content in the thick mix, which increased the resultant viscosity [11].

For the tear strength test, V-shaped specimens were used to standardize the tear location, and the thickness of the alginate specimens was 4mm, as the recommended thickness of the alginate impression was in the 4–6 mm range [16]. There was a significant difference in the tear strength between the two alginate materials used in this research when mixed as recommended by the manufacturers (groups 1 and 4). This finding was in agreement with Cohen B. I. et al., who evaluated the tear strength of four alginate impression materials mixed according to manufacturers’ instructions and found that there was a significant difference between them [16]. We observed that, in this previous study, the product that showed the highest tear strength was the one with the highest powder/water ratio, while there was no significant difference between the other three alginate materials mixed with the same powder/water ratio [16]. A similar finding was detected in the current investigation: that alginate mixed with a higher powder/water ratio (Tropicalgin) showed higher tear strength than that mixed with a lower powder/water ratio (Algistar). In addition, evaluating the effect of powder/water variation by 25% (which still gives a workable mix) on tear strength, direct proportionality between tear strength and powder/water ratio was also detected. This may be attributed to the higher filler content in the alginate mixed with a higher powder/water ratio, thus increasing the tear strength [4,17].

Regarding the detail reproduction, all the specimens reproduced the entire length of the 50 μm line and passed this test. This might be attributed to the sufficient flow of the alginate impression [18], even with a thick consistency. Guiraldo R. D. et al assessed the influence of alginate impression materials on the surface detail reproduction of stone models, and the result was that the 50 μm line was completely reproduced by all the tested alginate impression materials [5].

Various porosities and air bubbles were observed in the alginate mixed with a higher powder/water ratio (Figure 5b,d). This may be attributed to their higher viscosity entrapping more air bubbles, leading to more voids. It was reported in the literature that the viscosity of the impression material was one of the most important factors affecting bubble formation [19]. Decreasing the impression viscosity may increase its ability to flow into minute areas, resulting in fewer voids and less entrapment of air or saliva intraorally [19].

To perform this research clinically, in addition to the previous *in vitro* tests, clinical dental impressions were taken from volunteer dental patients. Interproximal contact areas were studied, as they are from the most critical sites due to their minimal thickness compared to the rest of the dental impression [20]. In general, no clear difference in tearing was clinically detected when examining dental impressions mixed with different powder/water ratios, i.e., the variation in the powder/water ratio did not clinically affect the tearing. Yet, it was observed that the tearing of the alginate was much affected by the thickness of the alginate due to the variation in the spaces between the teeth at interproximal contact areas.

It should be noted that the contact areas between the teeth could be proper or improper [21], as in the case of open contact [22] or excessive interproximal contact [23,24]. In patients with excessive interproximal dental contact areas, the impression materials in all tested groups did not enter between the teeth due to the intimate contact between them [21], preventing the impression from entering interdentally even if a lower powder/water ratio mix was used. It was noticed that the alginate impressions mixed with a lower powder/water ratio showed more flow in the area of buccal embrasures due to the lower viscosity, compared with those with a higher powder/water ratio.

Examining clinical impressions for patients with spaced teeth showed that the impression entered and exited the open contact without tearing. This might be attributed to the space between the teeth [21], which, in all groups, allowed for the entrance of enough thickness of the impression and its retrieval from the oral cavity without tearing.

Clinical impressions for patients with proper contact areas showed that all the impressions were subjected to tearing. This may be due to the low tear strength of this hydrocolloid alginate impression, accompanied by the reduced thickness at these interproximal areas [1]. Therefore, it could be postulated that there is a critical clinical value for alginate tearing, which is highly dependent upon its thickness.

It should be noted that there were differences between laboratory test outcomes and clinical results in regard to tear strength. Although *in vitro* measurements displayed that alginate mixed with higher powder/water ratios had a significantly higher tear strength compared with those mixed with lower powder/water ratios, clinically, no difference was detected. This might be attributed to the greater thickness of alginate used in *in vitro* tear strength tests (4 mm), which may accentuate the effect of increasing the powder content containing fillers and lead to a significant increase in tear strength.

It was observed that patients’ impressions mixed with higher powder/water ratios showed more voids than those with lower powder/water ratios. This may be due to the greater entrapment of saliva [25] and air [19] due to higher viscosity, as discussed previously. Although no obvious voids were detected in the *in vitro* alginate specimens mixed with lower powder/water ratios (Figure 5a,c), clear bubbles were clinically observed in these groups. This may be due to the presence of saliva that became trapped between the impression material and the surfaces of oral structures [25].

## 5. Conclusions

Although increasing the powder/water ratio of mixed alginate raised the resultant viscosity and tear strength by an *in vitro* test, no clear clinical difference in tearing was detected. The thickness of the alginate impression between adjacent teeth was a greater influencing factor, affecting tearing more than powder/water variation. Detail reproduction was minimally affected by variations in the powder/water ratio.

## Figures and Tables

**Table 1:** Materials used, their composition and mixing powder/water ratio as recommended by manufacturers.

Product	Algistar	Tropicalgin
Composition	Diatomaceous earth 40–50%, Cristobalite 15–20%, Potassium fluorotitanate 2–3%, Trisosodium phosphate 1–2%, Tetrasodium pyrophosphate 0.5–1%	Cristobalite 1–8%, Dipotassium hexafluototitanate 1–3%, Zinc oxide 0.5–2.5%, Phenolphthalein 0–0.2%, Diatomaceous earth.
Mixing powder/water ratio as recommended by manufacturer	19 g: 40 mL	18 g: 36 mL
Manufacturer	Müller-Omicron GmbH & Co. KG, Lindlar, Germany	Zhermack Spa, Italy

**Table 2:** Specimen grouping and powder/water ratio in each group.

Groups	Powder/Water Ratio
Group 1: Algistar (Manufacturer)	9 g: 40 mL
Group 2: Algistar (↑powder/water ratio than manufacturer)	23.75 g: 40 mL
Group 3: Tropicalgin (↓powder/water ratio than manufacturer)	13.5 g: 36 mL
Group 4: Tropicalgin (Manufacturer)	18 g: 36 mL

**Table 3:** Viscosity of the different groups (cp).

Groups	Viscosity (cp)	
	Mean	SD
Group 1: Algistar (Manufacturer)	654,000 ^a^	82
Group 2: Algistar (↑powder/water ratio than manufacturer)	2,152,000 ^c^	163
Group 3: Tropicalgin (↓powder/water ratio than manufacturer)	1,152,000 ^b^	122
Group 4: Tropicalgin (Manufacturer)	3,564,000 ^d^	180

Mean with different letters indicates statistically significance difference, *: significant (*p* < 0.05).

**Table 4:** Tear strength values of the different groups (N/mm).

Groups	Tear Strength (N/mm)	
	Mean	SD
Group 1: Algistar (Manufacturer)	0.83 ^a^	0.1
Group 2: Algistar (↑powder/water ratio than manufacturer)	1.34 ^c^	0.1
Group 3: Tropicalgin (↓powder/water ratio than manufacturer)	0.97 ^b^	0.1
Group 4: Tropicalgin (Manufacturer)	1.46 ^c^	0.1

Mean with different letters indicates statistically significance difference, *: significant (*p* < 0.05).

## Data Availability

The data presented in this study are available on request from the corresponding author.
